# Anxiety Assessment Using the Visual Analogue Scale Among Women Undergoing Elective Cesarean Section

**DOI:** 10.7759/cureus.79919

**Published:** 2025-03-02

**Authors:** Chalent Alexakis, Konstantinos Zacharis, Ismini Anagnostaki, Vasiliki Kalouda Tsapadikou, Spyridon Chondros, Sofia Kalantzi, Eleni Karakike, Stavros Kravvaritis, Theodoros Charitos

**Affiliations:** 1 Department of Obstetrics and Gynaecology, General Hospital of Lamia, Lamia, GRC

**Keywords:** elective c-section, pregnancy, secondary hospital, stress, visual analogue scale

## Abstract

Introduction

Patients scheduled to undergo surgery are likely to experience anxiety. Furthermore, anxiety levels are significantly higher in obstetric patients compared to the general population. The most common operation performed on obstetric patients is a cesarean section. The purpose of our study is to evaluate pregnant women's anxiety prior to an elective cesarean section.

Method

A prospective study was conducted on a sample of hospitalized pregnant women scheduled for elective cesarean section in a Greek secondary hospital from October 2023 to March 2024. An anonymous questionnaire was administered 12-15 hours before delivery. The first part of the questionnaire included demographic data and closed-ended questions regarding educational level, parity, type of anesthesia in a previous cesarean section, and the presence of pathology in the current pregnancy. The second part consisted of the Visual Analogue Scale (VAS) for anxiety assessment. A total of 50 pregnant women participated in this study with a mean age of 30.5 years old (SD=6.1).

Results

The average anxiety score was 6.14 with a standard deviation of 2.52. From the relationships examined, it emerged that higher VAS values are expected for younger women (r=-0.344; p=0.014). Additionally, significantly higher values were recorded for nulliparous women (M=7.54; SD=2.5) compared to multiparous women (M=5.65; SD=2.37) according to the independent samples t-test (t=-2.437; p=0.019). From the same analysis, significantly higher values were observed for pregnant women exhibiting pregnancy-related pathologies (M=7.60; SD=1.43) compared to women undergoing a smoothly progressing, uncomplicated pregnancy (M=5.78; SD=2.61) (t=-2.117; p=0.039). Finally, there was no statistically significant difference between the level of education or the type of anesthesia in a previous cesarean section and the VAS.

Conclusion

Anxiety prior to an elective cesarean section is expected to be greater in younger women and in nulliparous, while it is also higher for women with pregnancy-related pathologies. Participating in antenatal classes, creating a birth plan, and receiving professional support from health providers are examples of effective strategies to reduce anxiety, even before an elective cesarean section.

## Introduction

Anxiety is a distressing emotional response that impairs people's well-being and comfort. Preparing for a surgical operation is a common source of anxiety, as patients usually encounter uncertainty regarding various aspects of the procedure, such as anesthesia, hospitalization, and the possibility of surgical complications [1]. Although anxiety is a common reaction, it should not be ignored, as high levels of stress are likely to negatively affect the outcome of the surgery [2]. The intensity of anxiety experienced by patients undergoing surgery varies. There have been attempts to correlate preoperative anxiety with factors that are potentially more stressful, as well as with factors that have a positive effect on the patient. Preoperative anxiety affects approximately 60-80% of patients undergoing surgery in Western societies [3,4]. It is likely that the main factors that influence its intensity are gender, age, type of surgery, previous experience with hospitalization and surgery, the character of the individual, the patient's education, and information [5,6]. Further research has attempted to illustrate the mechanisms through which anxiety can influence surgical outcomes, but more investigation is required to understand the underlying process.

Preoperative anxiety is a subject that has been thoroughly researched in various other surgical interventions; however, its effects on the cesarean section (CS) experience have not been fully investigated, especially in terms of differences between elective and emergency CS or in specific populations, despite the issue being identified [7]. This is especially significant because it has been proposed that a major factor in women's requests for elective CS is their fear of labor. Compared to other surgical interventions, preoperative anxiety during cesarean deliveries is higher, particularly in developing nations [8]. According to limited data in the literature, preoperative anxiety affects between 73.3% and 86% of women undergoing delivery via CS [9].

According to available data, the rate of cesarean births in Greece is particularly high. Specifically, in the four-year period from 2016 to 2020, there was a 56.4% increase in CS, with the method being chosen for half of all births, far exceeding the World Health Organization's recommendation of 10-15% of all births [10,11]. At the same time, studies have shown that women who undergo CS can experience psychological symptoms. This phenomenon has caused concerns regarding the psychological effects of CS on maternal well-being, in addition to its medical indications.

Some studies identify the psychological effects of CS, namely, postpartum depression and anxiety. Current studies suggest that women who undergo CS have increased levels of anxiety preoperatively and postpartum [12,13]. Anxiety prior to a CS is most commonly influenced by concerns regarding the procedure itself, anesthesia, and maternal and fetal complications. Moreover, women with inadequate information or poor surgical preparation would be identified as more anxious [14]. These findings highlight the importance of individual psychological treatment and antenatal education for women undergoing CS.

Despite all the studies on preoperative anxiety in surgical patients, there are not many studies focused on CS. Nevertheless, available data indicate that pre-CS anxiety is significantly higher than in other surgeries, particularly in primiparous women and pregnant women with complications [15]. The relationship between high CS rates and the necessity to address maternal anxiety is of the highest relevance. The increased popularity of elective cesarean delivery, frequently driven by labor fear, even more emphatically calls for effective prenatal counseling and psychiatric treatment [16].

Given the limited studies in Greece regarding preoperative maternal anxiety before an elective CS, this study aims to evaluate anxiety levels and identify potential associating factors (age, parity, pregnancy-related pathologies, etc.). We hypothesize that younger women, nulliparous women, and those with pregnancy complications will report higher anxiety levels. This was accomplished using the Visual Analogue Scale (VAS), a widely used tool for assessing subjective anxiety levels in clinical settings. The VAS was selected due to its ease of use, simplicity, and capacity to quantify anxiety levels precisely in real time. Because the VAS enables a quick assessment, it is specifically suitable for the busy preoperative obstetric setting. This is in contrast to more sophisticated tools like the State-Trait Anxiety Inventory (STAI) or the Hospital Anxiety and Depression Scale (HADS), which require more time and mental effort from participants.

## Materials and methods

Study design, area, and period

A prospective study was conducted on a sample of hospitalized pregnant women scheduled to undergo a planned CS at the maternity ward of the General Hospital of Lamia during the period from October 12, 2023, to March 31, 2024, after obtaining approval from the institute's Scientific Council (approval number: Σ/17759/27-09-2023). The sample consisted of 50 women. The hospital is a secondary care facility located in Lamia, approximately 210 kilometers from Athens. It handles approximately 350 deliveries annually.

Inclusion criteria and exclusion criteria

The study included all women who delivered via elective CS while excluding those who underwent emergency cesarean delivery, patients using anxiolytics, and those with a language barrier. In order to avoid any potential confounding effects, women who were using anxiolytic drugs were not included. This is because these medications have the ability to drastically change anxiety levels and mask correlations with clinical and demographic factors.

Questionnaire validation and standardization

The VAS, a well-used and validated instrument in psychological and medical research, was part of the questionnaire used in this study to measure anxiety. It has been widely used to measure preoperative anxiety in the past, yielding consistent and repeatable results in a variety of populations. The questionnaire was given to each respondent in a controlled hospital environment under comparable circumstances, and all participants got brief instructions on how to use the scale consistently to guarantee response standardization. These actions were taken in an effort to reduce bias and improve the accuracy of the information gathered.

Data collection instrument and procedures

An anonymous questionnaire (Table 1) was distributed 12-15 hours prior to the scheduled CS. The first part of the questionnaire included demographic data and closed-ended questions, while the second part consisted of the VAS for the assessment of anxiety. The sample included 50 women aged 17-45 years (M=30.5; SD=6.1) for whom the level of anxiety on the VAS was recorded as well as the educational level, the parity, the type of anesthesia in a previous CS, and the existence of pathology in the current pregnancy, such as gestational diabetes (GDM), preeclampsia, or bleeding during the course of pregnancy.

**Table 1:** Questionnaire used to evaluate preoperative anxiety in women undergoing elective cesarean section Data in the table: Ν(%) categorical variables and mean±SD continuous variables

Variables	Categories	N	%
Education level	Primary school	7	14
	Junior high school	6	12
	High school	20	40
	University/college graduate	14	28
	Master's/PhD holder	3	6
Parity	Nulliparous (0 previous births)	13	26
	Multiparous (≥1 previous births)	37	74
Previous cesarean section	No	14	28
	Yes, general anesthesia	5	10
	Yes, regional anesthesia	31	62
Pregnancy pathology	Yes	10	20
	No	40	80
Anxiety score (Visual Analogue Scale)	Mean±SD	6.14±2.52	-

Data processing and analysis

To examine the correlation between the VAS and the age of the participants, Pearson's correlation was used. The effect of education level on the VAS was assessed using analysis of variance (ANOVA). Additionally, t-tests for two independent samples were conducted to assess the differences in the scale based on previous delivery, the use of anesthesia in a prior CS, and the presence of pathology. The analysis was performed using IBM SPSS Statistics for Windows, Version 28.0 (Released 2021; IBM Corp., Armonk, New York, United States), and the significance level was set at 0.05 for all cases.

## Results

**Figure 1:**
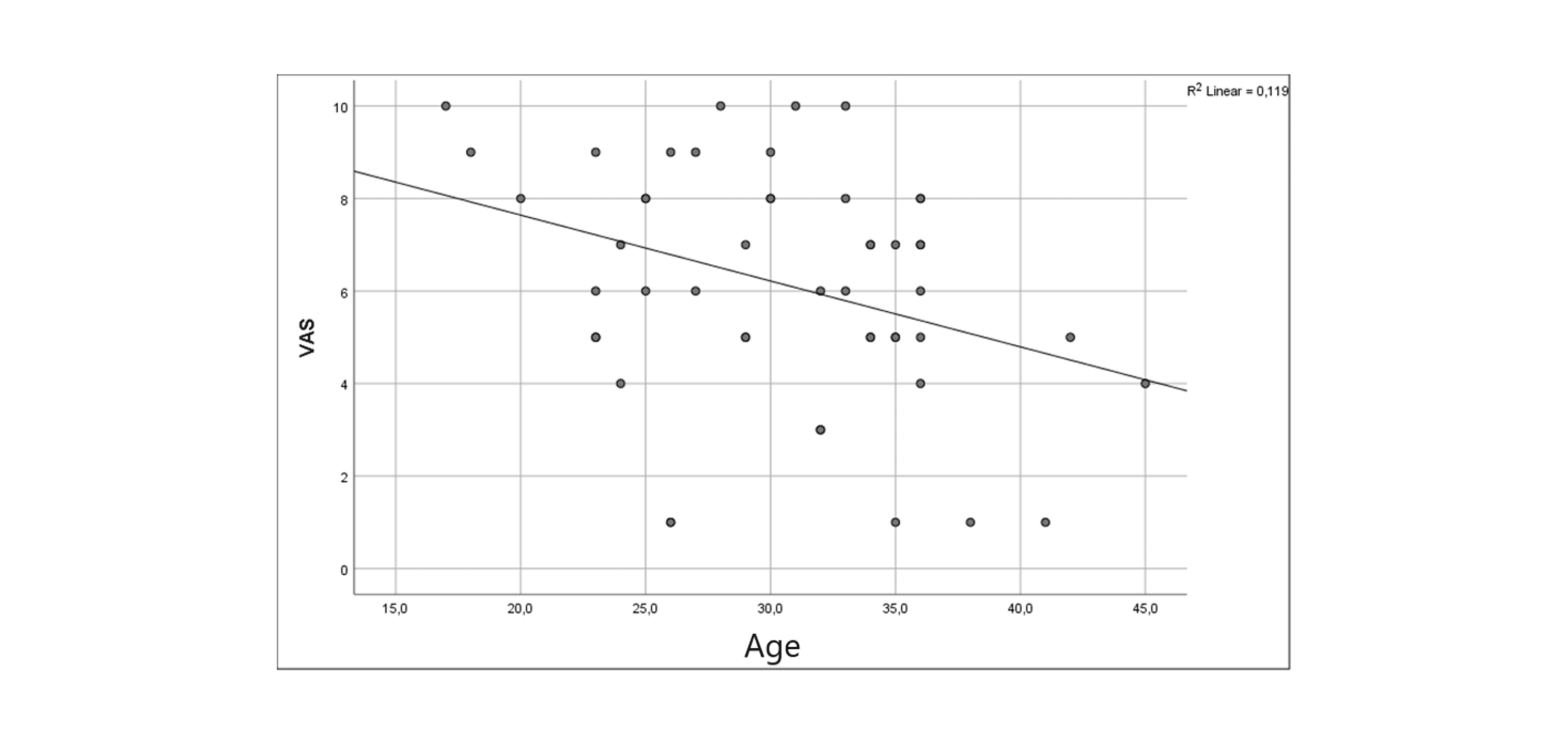
The association of the Visual Analogue Scale with the age of the sample

Table 2 presents the effect of participants' age on the VAS, which assesses the anxiety of hospitalized pregnant women before a planned CS. Specifically, it is observed that there is a statistically significant negative correlation between the VAS and age (r=-0.344). Therefore, higher age values are expected to be associated with lower values on the VAS. The correlation is statistically significant because the p-value is less than 0.05 (p=0.014). The statistically significant correlation is depicted in Figure 1.

**Table 2:** Correlation between the anxiety level (Visual Analogue Scale) and the age of the sample Pearson's correlation is used to evaluate the correlation between age and anxiety level. Significance threshold: p<0.05

Variable	Pearson's r	Test statistic (t-value)	P-value
Age vs. anxiety score (Visual Analogue Scale)	-0.344	t(48)=-2.56	0.014

In Table 3, the effect of the educational level of the sample on the VAS is presented. Specifically, it is observed that there is no statistically significant difference between the level of education and the VAS, as the p-value is greater than 0.05 (p=0.534) and the statistical function is F(4,45)=0.796.

**Table 3:** Effect of education level on anxiety scores (Visual Analogue Scale) Data are presented as mean±SD. Analysis of variance was performed to compare groups. Significance threshold: p<0.05

Education level	N	Mean value	Standard deviation	95% CI (lower limit)	95% CI (upper limit)	F-value	P-value
Primary school graduate	7	6.86	2.11	4.90	8.81	0.796	0.534
Junior high school graduate	6	7.33	1.50	5.75	8.91		
High school graduate	20	5.85	2.92	4.48	7.22		
University/college graduate	14	6.00	2.32	4.66	7.34		
MSc holder	3	4.67	3.21	-3.32	12.65		
Total	50	6.14	2.52	5.42	6.86		

**Figure 2:**
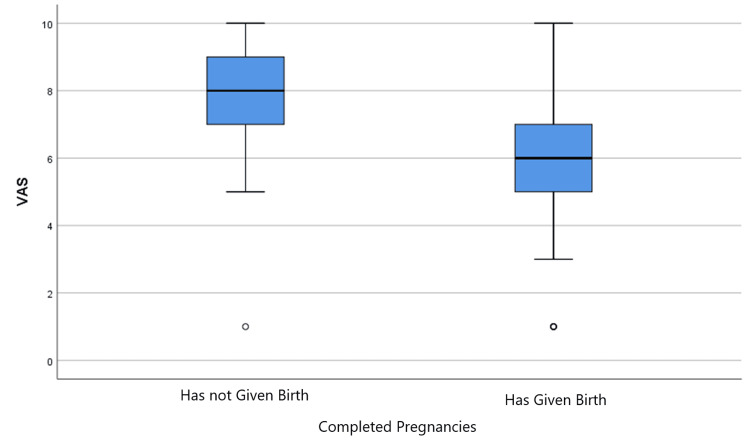
Effect of previous childbirths on the Visual Analogue Scale

Table 4 presents the effect of previous childbirth experience in the sample concerning the VAS. Specifically, it is observed that there is a statistically significant difference between them, as the p-value is less than 0.05 (p=0.019) and the statistical function is t(48)=-2.437. The statistically significant difference is illustrated in Figure 2.

**Table 4:** Effect of previous childbirth on anxiety score (Visual Analogue Scale) Data are presented as mean±SD. Independent samples t-test was performed. Significance threshold: p<0.05

Parity	N	Mean±SD	Test statistic (t-value)	P-value
Primiparous/multiparous	37	7.54±2.50	t(48)=-2.437	0.019
Nulliparous	13	5.65±2.37		

Table 5 presents the effect of the type of anesthesia used in a previous CS on the VAS. Specifically, it is observed that there is no statistically significant difference between the type of anesthesia and the VAS, as the p-value is greater than 0.05 (p=0.884) and the statistical function is t(34)=-0.147.

**Table 5:** Effect of type of anesthesia in previous cesarean section on the Visual Analogue Scale Data are presented as mean±SD. Independent samples t-test was performed. Significance threshold: p<0.05

Type of anesthesia in previous cesarean section	N	Mean±SD	Test statistic (t-value)	P-value
General anesthesia	5	5.60±0.54	t(34)=-0.147	0.884
Regional anesthesia	31	5.68±2.58		

**Figure 3:**
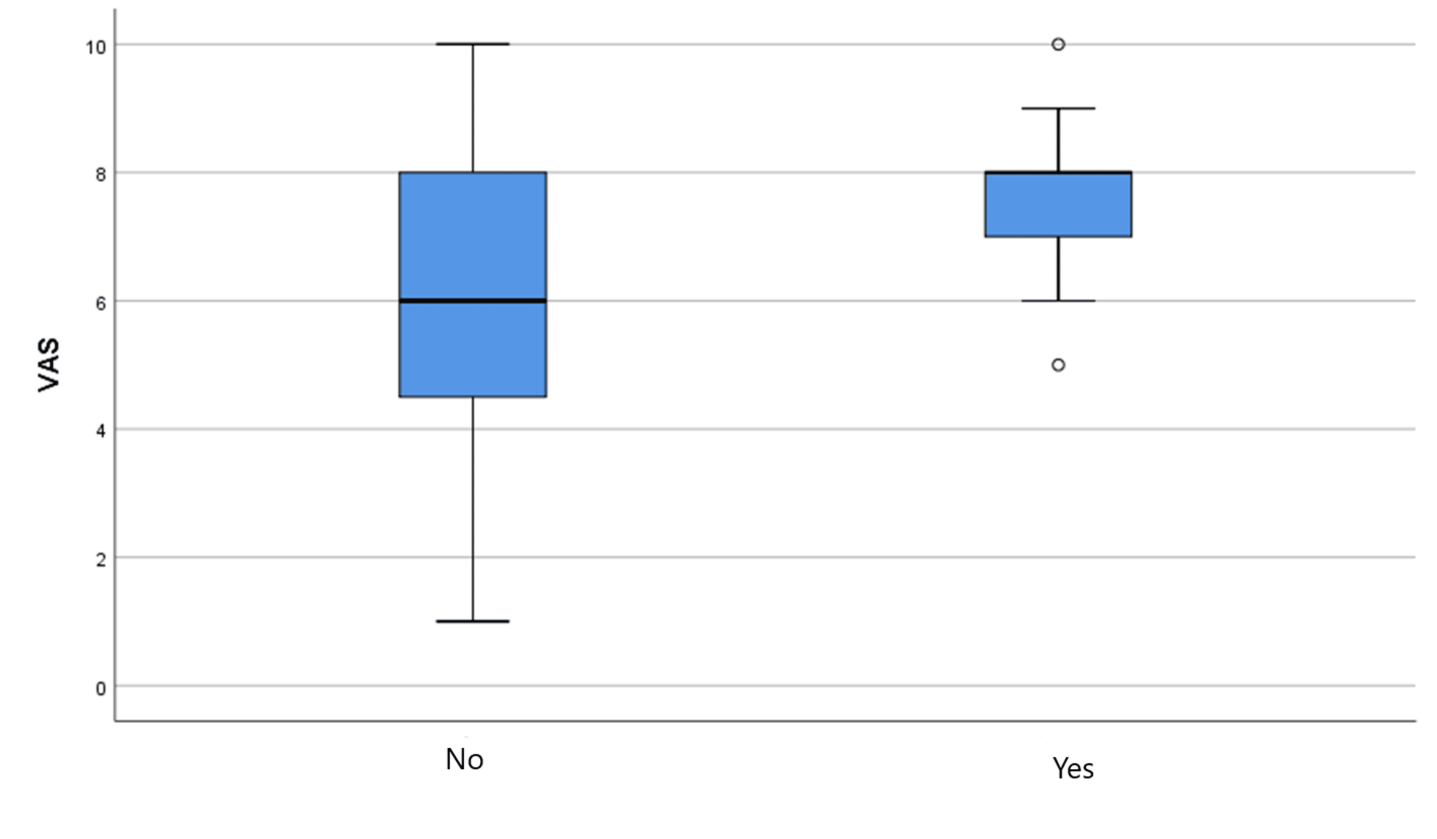
Effect of the presence of pathology on the Visual Analogue Scale

Table 6 presents the effect of the presence of a pathology in the current pregnancy on the VAS. Specifically, it is observed that there is a statistically significant difference between the presence of pathology and the VAS, as the p-value is less than 0.05 (p=0.039) and the statistical function is t(48)=2.117. The statistically significant differentiation is illustrated in Figure 3.

**Table 6:** Effect of the presence of pathology on the Visual Analogue Scale Data are presented as mean±SD. Independent samples t-test was performed. Significance threshold: p<0.05

Presence of pathology in current pregnancy	N	Mean±SD	Test statistic (t-value)	P-value
Yes	10	7.60±1.43	t(48)=2.117	0.039
No	40	5.78±2.61		

## Discussion

The study's findings show how nervous expectant mothers are before an elective CS. The results are consistent with other research, including that of Hepp et al., which highlighted the psychological challenges associated with cesarean delivery and looked at the course of anxiety in women who gave birth via this procedure [17]. Our research provides crucial information regarding the mental state of women planning elective CS by delving deeper into preoperative anxiety-causing factors, including age, prior delivery experience, and the existence of pregnancy-related diseases. The current study also highlights the need for implementing strategies aimed at reducing anxiety, which has also been supported by other studies [12].

Age and anxiety levels as determined by the VAS showed a statistically significant negative correlation in our study. Compared to older women, younger women tended to report higher levels of stress. This finding is in line with research by Ayers et al., who found that younger women were more likely to experience anxiety because they were less experienced and hesitant about undergoing surgery [18]. Interventions aimed at reducing anxiety should target younger women specifically, offering them more information and support to alleviate their worries.

Additionally, compared to women who had previously given birth, those who had never given birth before (primiparous) displayed higher anxiety levels. This result is consistent with the study by Saisto and Halmesmäki, who claimed that anxiety levels can rise due to a lack of experience and fear of childbirth, particularly in women having CS [16]. Furthermore, primiparous women having an elective CS typically have higher anxiety levels because they are afraid of the procedure and are unclear about it, according to several studies [13,19]. Organizing a birth plan and participating in parental education programs can assist primiparous women feel less nervous and manage childbirth better.

A correlation was found between elevated anxiety levels and pregnancy-related pathologies like preeclampsia and GDM. This finding is in line with a 2022 study by Zhang et al. that found pregnant women who experience pregnancy-related problems are more likely to experience anxiety because they are uncertain about the outcome of their pregnancy and birth [15]. Additionally, the study by Van der Gucht and Lewis clarified that pregnant women who have disorders experience additional psychological strain, which could make them feel more anxious [20]. For women with disorders, information and psychological support are crucial to anxiety management.

Even though there isn't a significant statistical correlation between anxiety levels and educational level, women's knowledge and information are important for reducing stress levels. Women can feel more prepared and less nervous if they take part in educational programs that highlight the CS process and recovery techniques [14]. Research indicates that education and psychological assistance can significantly improve women's psychological well-being before giving birth [21].

Giving women ongoing care and information during the CS procedure may lead to a more positive birth experience, according to a research by Hepp et al. [17]. Furthermore, Fenwick et al. found that emotional support from family and partners is extremely important in reducing women's stress levels [22]. In order to minimize stress and enhance outcomes for both the mother and the newborn, health practitioners should actively involve women in the decision-making process and encourage them to voice their concerns.

A constraint of our research is the limited sample size, which could potentially impact the findings' generalizability. Furthermore, just one center was used for the study; thus, local influences can have an impact on our findings. Because of this, it might not be as applicable to larger populations with a wider range of demographic traits. To enhance the robustness of a potential comparable study, multicenter research with a larger sample size could be useful for deriving stronger conclusions. A comparative study between women undergoing educational sessions before an elective CS and a control group could provide a more comprehensive evaluation of the intervention's effectiveness.

Our study's dependence on self-reported anxiety levels, which could result in self-report bias, is another possible drawback. Individuals' perceptions of anxiety might differ greatly depending on their personality, past experiences, and outside factors like the hospital setting or interactions with medical personnel. Furthermore, some participants may have overreported or underreported their anxiety levels due to social desirability bias. To get a more objective assessment, future research may add physiological markers of worry, like cortisol levels or heart rate variability, to self-reported assessments.

Finally, in our study, there was no correlation between anxiety levels and educational level. Although the literature indicates that higher education is often linked with lower levels of anxiety due to improved coping strategies and information availability, other factors such as personality traits, previous childbirth experiences, and emotional support networks might play a more significant role [14]. Since all participants in our study received the same healthcare information, it is also possible that anxiety was not significantly influenced by educational level. This discrepancy should be further explored in future studies by considering additional psychological factors.

## Conclusions

Our study highlights the importance of psychological support for women planning a cesarean delivery. Anxiety is a serious issue that could negatively affect a woman's experience throughout the procedure. Therefore, anxiety-reduction strategies, attending parenting classes, creating a birth plan, and seeking medical assistance are all necessary. These methods can significantly improve women's experiences and lead to better physical and psychological results.
